# Effect of weekend admission on in-hospital mortality and functional outcomes for patients with acute subarachnoid haemorrhage (SAH)

**DOI:** 10.1007/s00701-016-2746-z

**Published:** 2016-03-01

**Authors:** Harshal Deshmukh, Matthew Hinkley, Louise Dulhanty, Hiren C. Patel, J. P. Galea

**Affiliations:** Newcastle University and James Cook University Hospital, Middlesbrough, TS4 3RP UK; Salford Royal Foundation Trust, Manchester Academic Health Sciences Centre, Stott Lane, Salford, M6 8HD UK; Ninewells Hospital and Medical School, Ninewells, Dundee, DD1 9SY Scotland UK

**Keywords:** SAH, Weekend effect, Mortality due to SAH, Survival in SAH

## Abstract

**Background:**

Aneurysmal subarachnoid haemorrhage (aSAH) is an acute cerebrovascular event with high socioeconomic impact as it tends to affect younger patients. The recent NCEPOD study looking into management of aSAH has recommended that neurovascular units in the United Kingdom should aim to secure cerebral aneurysms within 48 h and that delays because of weekend admissions can increase the mortality and morbidity attributed to aSAH.

**Method:**

We used data from a prospective audit of aSAH patients admitted between January 2009 and December 2011. The baseline demographic and clinical features of the weekend and weekday groups were compared using the chi-squared test and *T*-test. Cox proportional hazards models (Proc Phreg in SAS) were used to calculate the adjusted overall hazard of in-hospital death associated with admission on weekend, adjusting for age, sex, baseline WFNS grade, type of treatment received and time from scan to treatment. Sliding dichotomy analysis was used to estimate the difference in outcomes after SAH at 3 months in weekend and weekday admissions.

**Results:**

Those admitted on weekends had a significantly higher scan to treatment time (83.05 ± 83.4 h vs 40.4 ± 53.4 h, *P* < 0.0001) and admission to treatment (71.59 ± 79.8 h vs 27.5 ± 44.3 h, *P* < 0.0001) time. After adjustments for adjusted for relevant covariates weekend admission was statistically significantly associated with excess in-hospital mortality (HR = 2.1, CL [1.13–4.0], *P* = 0.01). After adjustments for all the baseline covariates, the sliding dichotomy analysis did not show effects of weekend admission on long-term outcomes on the good, intermediate and worst prognostic bands.

**Conclusions:**

This study provides important data showing excess in-hospital mortality of patients with SAH on weekend admissions served by the United Kingdom’s National Health Service.; However, there were no effects of weekend admission on long-term outcomes.

**Electronic supplementary material:**

The online version of this article (doi:10.1007/s00701-016-2746-z) contains supplementary material, which is available to authorized users.

## Introduction

Aneurysmal subarachnoid haemorrhage (aSAH) is an acute cerebrovascular event with an incidence of 9 per 100,000 person years [7]. Although less common than other forms of ischaemic and haemorrhagic stroke, it has a higher socioeconomic impact as it tends to affect younger patients in their working life [20]. aSAH has a very high (up to 50 %) mortality rate and it is estimated that less than 60 % of survivors return to a functionally independent life [21]. The main complications in the acute phase following SAH that are associated with increased morbidity and mortality are hydrocephalus, cerebral vasospasm and aneurysmal re-bleeding (80 % mortality [19]). In the prospective Cooperative Aneurysm Study, re-bleeding was maximal (4 %) on the 1st day after SAH and then constant at a rate of 1–2 % per day over the subsequent 4 weeks [11]. The risk of both cerebral vasospasm and delayed cerebral ischaemia (DCI) is highest between the 3rd and 10th day following ictus. While historically, patients presenting after 3 days of ictus were not offered aneurysm treatment due to the heightened risk of spasm, better medical management of DCI, as well as a wider repertoire of intervention (medical and surgical) to counteract vasospasm during endovascular procedures, has meant that attempts at endovascular treatment of aneurysms beyond the 3-day golden period is now becoming more accepted practice. This is of particular significance in an era that has seen a move towards centralisation of specialist neurovascular centres and inevitable longer transit times to centres for definitive neurovascular care.

The recent NCEPOD study into management of aSAH has recommended that neurovascular units in the United Kingdom should aim to secure cerebral aneurysms within 48 h from ictus [1]. This is a significant decrease in the 72-h time window recommended by the European Stroke Organisation Guidelines [16]. This new target also raises significant issues with respect to both human and financial resources: provision of neurovascular surgeons, neuro-interventional radiologists, specialist radiographers, specialist neuro-anaesthetic staff and trained theatre/angio-suite support services. While the NCEPOD report looked at the patient journey of a national cohort of 400 patients, it is unclear on what basis this recommendation was made. While several studies have shown a detrimental effect of weekend admission on mortality after myocardial infarction, pulmonary embolism and stroke, the same effect was not demonstrated in patients with SAH in two independent studies [6, 22]. The findings of both these studies should be interpreted with caution, as these studies did not adjust for the severity of SAH at baseline (WFNS grade) and treatment given. They also did not investigate the effect on long-term functional outcomes in patients with SAH.

It is, therefore, yet unknown whether a limited delay in securing aneurysms following aSAH leads to a poorer outcome. This remains an important unanswered question, given the potential financial and human resource repercussions that would ensue should the NCEPOD recommendations be followed in this time of financial crisis for the National Health Service (NHS). The purpose of this study is to investigate the effect of weekend admission on in-hospital mortality and 30-day mortality on patients with aSAH, as well as long-term functional outcomes in patients with aSAH.

## Methods

### Study population

This study represents a prospective audit of acute SAH patients admitted between January 2009 and December 2011. Our data represented a catchment population of 5 million, from a geographical area in the northwest of England, with admissions from 12 large regional hospitals. Weekend admission was defined as the time from 16.00 h on Friday to 16.00 h on Sunday. Patients with SAH who were admitted on bank-holidays were categorised in the weekend group using similar time cut-offs. Data were collected for age at admission, gender, baseline WFNS grade World Federation of Neurosurgical Societies (WFNS) Grade, treatment modalities following admission, time from scan to admission and then from admission to treatment. Length of stay in hospital was measured along with deaths during in-hospital admission and at the end of 3 months. Functional outcome at the end of 3-month periods was estimated using the ordinal Glasgow Outcome Scale (GOS).

### Statistical analysis

The baseline demographic and clinical features of the weekend and weekday groups were compared using the chi-squared test and *T*-test. Cox proportional hazards models (Proc Phreg in SAS) were used to calculate the adjusted overall hazard of in-hospital death associated with admission on weekend, adjusting for age, sex, baseline WFNS grade, type of treatment received and time from scan to treatment. Cox-proportional hazard models were also used to calculate the adjusted overall hazard of death at discharge (defined as in-hospital mortality) and a poor functional outcome after discharge from hospital and at the end of 3 months’ follow-up period measured with GOS. To enhance the statistical power, we utilised a sliding dichotomy approach to study the weekend effect on GOS at 3 months. The sliding dichotomy also dichotomises outcome into a binary measure, in which the cut point defining favourable or unfavourable outcome depends on the predicted prognosis for an individual patient on entry into the study. It is considered to be more informative and statistically powerful than a simple dichotomous outcome (and Cox Proportional Hazard models) [14]. For sliding dichotomy, we first estimated the baseline prognostic risk in each patient by calculating C-statistics of regression model predicting the outcome (GOS 1 + 2 vs GOS 3 + 4 + 5) in the overall dataset (goodness of fit of logistic regression models) using all the baseline covariates mentioned above. The estimated C-statistics for all the baseline variables was 0.80 while that for baseline WFNS was 0.77. For the easy interpretation of results, we then classified the patients into three prognostic groups. WFNS I as “good”, WFNS II and III as “intermediate” and WFNS IV and V as “worst”. The effect of weekend treatment on these newly generated dichotomous outcomes was then estimated using binary logistic regression, with stratification by prognostic bands and adjustment for the baseline covariates mentioned above.

## Results

### Demographic characteristics of the study population

Table 1 shows the demographic characteristics of the study population. During the period of 2009 to 2011, the study population consisted of 285 patients with SAH admitted to hospital on weekdays and 100 patients admitted to hospital on weekends. There was no statistical difference in the mean age (53 ± 12 vs 53 ± 13), gender (27 % females vs 37 % females) and WFNS grades (good WFNS grades I + II, 72 vs 74 %, and bad grades III + IV, 28 vs 26 %) in patients admitted on weekdays and those admitted on weekends. There was no statistically significant difference in treatment following the SAH in patients admitted on weekdays and those admitted on weekends.

**Table 1:** Demographic and clinical characteristics of the study population

	WEEKDAY (*n* = 285)	WEEKEND (*n* = 100)	*P* value
Age (mean ± SD)	53 ± 12	53 ± 13	0.84
Female patients (%)	77 (27 %)	37 (37 %)	0.07
WFNS grade			
I	156 (54 %)	55 (54 %)	0.85
II	53 (18 %)	21 (20 %	
III	9 (3 %)	2 (2 %)	
IV	30 (11 %)	8 (8 %)	
V	36 (13 %)	15 (15 %)	
Treatment			
No treatment (%, *n*)	10 (3.5 %)	8 (8 %)	0.1
Endovascular (%, *n*)	238 (83.5 %)	72 (72 %)	
Surgical (%, *n*)	37 (13 %)	20 (20 %)	
Mean scan to admission time	14.05 (34.5)	10.7 (20.8)	0.38
Mean admission to treatment time	27.5 (44.3)	71.59 (79.8)	<0.0001
Mean scan to treatment time	40.4 (53.4)	83.05 (83.4)	<0.0001
Mean length of stay in hospital	19.9 (17.8)	20.5 (19.57)	0.76
In-hospital death after admission	27 (9.5 %)	16 (16 %)	0.08
Re-bleeds	5 (1.7 %)	0 (0 %)	
Death at 3 months	29 (10.2)	17 (16.8)	0.08
GOS at 3 months			
GOS 1 & 2	30 (10.5 %)	18 (17.8 %)	0.14
GOS 3 & 4	52 (18.3 %)	15 (14.8 %)	
GOS 5	202 (71.13 %)	68 (67.3 %)	

After the symptomatic presentation, patients firstly underwent a scan, followed by admission and then treatment. There was no statistically significant difference in scan to admission time (14.05 ± 34.5 h vs 10.7 ± 20.8 h, *P* = 0.38) in SAH patients treated on weekends and those treated on weekdays. However, those admitted on weekends had a significantly higher scan to treatment time (83.05 ± 83.4 h vs 40.4 ± 53.4 h, *P* < 0.0001) and admission to treatment (71.59 ± 79.8 h vs 27.5 ± 44.3 h, *P* < 0.0001) time. There was no significant difference in the overall length of stay in hospital (20.5 ± 19.57 days vs 19.9 ± 17.8 days, *P* = 0.76) in the weekend and weekday groups. In a univariate analysis there were no statistically significant differences in length of hospital stay (19.9 ± 17.8 vs 20.5 ± 19.57, *P* = 0.76) and in-hospital deaths (27 ± 9.5 % vs 16 ± 16 %, *P* = 0.08). There was no significant difference between GOS at 3 months between the weekday (GOS 1 & 2 = 10.5 %, GOS 3 & 4 = 18.31 % and GOS 5 = 71.13 %) and weekend groups (GOS 1 & 2 = 17.82 %, GOS 3 & 4 = 14.85 % and GOS 5 = 67.33 %) (*P* = 0.14).

### Weekend admissions and all-cause mortality and functional outcomes in SAH

**Fig. 1:**
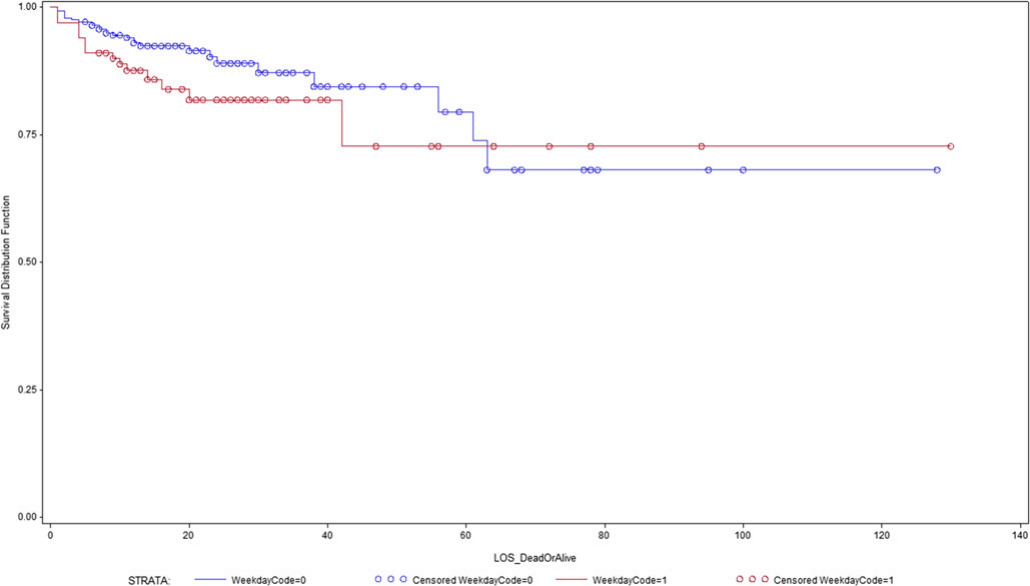
Weekend effect in survival at discharge of patients with SAH. Cox propotional hazard models showing effect of weekend admission and risk of in-hospital mortality in patients with acute SAH (Blue colour = 0 = weekday; red colour = 1 = weekend)

Figure 1 shows the Kaplan-Meier survival analysis curve. Table 2 shows the hazard ratios from Cox proportional hazard models for weekend admission on in-hospital mortality and GOS at the end of 3 months. After adjustments for adjusted for age, sex, WFNS grade at baseline and treatment modality, weekend admission was statistically significantly associated with in-hospital mortality (HR = 2.1, CL [1.13–4.0], *P* = 0.01). This weekend effect on excess mortality persisted after adjustment for time from scan to treatment (HR = 2.1, CL [1.13–4.0], *P* = 0.01), thereby implying that the excess weekend mortality cannot be completely explained by delay in treatment following scan and admission in the hospital.

**Table 2:** Cox proportional hazard models showing the effect of weekend admission on survival at discharge and GOS at 3 and 6 months

Outcome	Hazard ratio	CL	*P* value
Mortality at discharge^a^	2.1	1.13–4.0	0.01
Mortality at discharge adjusted for time from scan to treatment^b^	2.95	1.08–7.91	0.03
GOS at 3 months^c^	1.6	0.70–3.9	0.24

^a^Adjusted for age, sex, WFNS grade and treatment

^b^Adjusted for age, sex, WFNS grade, treatment type and scan to treatment time

^c^Logistic regression analysis comparing grades 4 and 5 versus grades 1, 2 and 3. Adjusted for age, sex, WFNS grade, treatment and scan to treatment type

### Weekend admissions and functional outcomes using sliding dichotomy analysis

Table 3 shows the effect of weekend admission from the sliding dichotomy analysis on functional outcomes in SAH patients. Outcomes were dichotomised as “better than expected” and “worse than expected” in each of the three prognostic bands. After adjustments for all the relevant baseline covariates, there are no effects of weekend admission on the good (OR = 0.59, CL [0.22, 1.57], *P* = 0.27), intermediate (OR = 1.03, CL [0.25–4.25], *P* = 0.96), and worst (OR = 0.49, CL [0.18–1.45], *P* = 0.17) prognostic bands.

**Table 3:** Sliding dichotomy analysis of weekend effect for GOS at 3 months after SAH

							Worse than expected	Better than expected	Odds ratio	*P* value
Prognosis^a^	GOS	1	2	3	4	5				
Good (WFNS I)	Weekend	2	0	2	11	141	15	141	0.59 (0.22, 1.57)	0.27
	Weekday	5	0	0	3	47	8	47		
Intermediate (WFNS II & II)	Weekend	8	0	1	12	41	9	53	1.03 (0.25, 4.25)	0.96
	Weekday	2	0	2	4	15	4	19		
Worst prognosis (WFNS IV & V)	Weekend	19	1	11	15	20	20	46	0.49 (0.18, 1.45)	0.17
	Weekday	10	1	3	3	6	11	12		

^a^Good, worse and intermediate prognosis bands were based on WFNS cut-offs

## Discussion

We report results of a prospective cohort study indicating that admission with SAH at weekend is associated with significantly higher in-hospital mortality after adjustments of all the relevant clinical covariates. Our study did not find any effect of weekend admissions on long term outcomes in patients with SAH.

There are several studies describing a wide variety of diagnoses that have demonstrated a “weekend effect” on mortality in patients who are admitted in hospital during weekends and holidays. Bell and Redelmeier [4] reported one of the biggest studies looking at the weekend-effect on mortality on 4 million patients and showed increased mortality on a wide variety of conditions, such as pulmonary embolism and ruptured aortic aneurysm. However, the study looked at effect of weekend admissions on 100 different conditions and hence the estimates should be interpreted with caution as the authors did not sufficiently adjust for the multiple hypotheses tested. Since then, studies have looked for specific conditions such as stroke [2, 3, 13], acute myocardial infarction [9, 12], pulmonary embolism [8], metastatic cancer [15], gastro-intestinal bleeding [18], hip fractures [17] and intensive care unit admission [10]. The results from these studies varied, with some demonstrating higher mortality associated with weekend admissions and others showing no effect—this is not surprising, given that they were performed in different healthcare settings. Our literature search showed two studies looking at the effect of weekend admissions on patients with SAH: one study on a United States population [6] and another on a Chinese population [22]. However, as mentioned above, none of these adjusted for important baseline characteristics, such as severity of SAH, and did not look at functional outcomes, hence their results should be interpreted with caution. Furthermore, there is no study in the setting of the United Kingdom’s NHS looking at the effect of weekend admissions with SAH. Our study shows that weekend admission was associated with poorer access to healthcare; i.e. increased time from a diagnostic scan to treatment and increased in-hospital mortality on those admitted with SAH on weekend. Interestingly, the excess in-hospital mortality with weekend admissions was not completely explained by the delays in access to treatment, indicating that there are can be factors at play which can explain the excess mortality. We also looked at the effect of weekend admissions on long-term outcomes (3 months) by using two statistical approaches. First, we used a binary classification system comparing good versus the poor outcomes and, second, we did a sliding dichotomy analysis, which can provide increased power in analysis of ordinal outcomes variables [14]. Both analyses did not show any effect of weekend admissions on long-term outcomes of the patients admitted with SAH. Although the weekend admission was associated with excess mortality at discharge, it was not associated with worse outcomes at 3 months. This can be explained by two factors: (1) After the discharge following aSAH, good clinical recovery happened in both the weekend and weekday subgroups. For example, in those with the worst prognostic bands, 60 % of those admitted on weekdays had better than expected outcomes, while 52 % of those admitted on weekends had better than expected outcomes. This led to dilution of the weekend effect seen at discharge. (2) These dichotomous analyses are less powerful than time to event analysis looking at mortality at discharge.

In general, it is assumed that increased mortality is associated with poor treatment, and some studies have demonstrated a relationship with reduced nursing numbers or poor availability of senior medical staff at weekends. In our centre, during the time of this study, there may have been delays in confirming that the SAH was due to an aneurysm pathology, there were no specialist neurovascular rounds, lower numbers of senior Intensive Care Unit medics, lower availability of neurosurgeons with a vascular interest and poorer access to secure aneurysms. While most of these factors may have contributed, none of our patients experienced re-bleeding at the weekend (re-bleed rate of 1.3 %), and therefore time to treating the aneurysm was not responsible for the difference in mortality. There are minimal pan-European data available to compare the treatment time following SAH. However, there is some evidence to suggest that the treatment time could vary according to geographical locations across Europe [5] . It is interesting to note that in the National Confidential Enquiry into Patient Outcome and Death (NCEPOD) United Kingdom data, only 70 % had their aneurysm treated within 24 h following admission. The NCEPOD report in 2013 highlighted some of the reasons for this delay: more than 60 % of neurosurgical centres in United Kingdom do not have an interventional radiologist available 7 days a week (service infrastructure availability is much less), 80 % do not have a policy defining optimal timing of treatment of SAH patients and 90 % of all hospitals do not have access to computed tomography scanning 24 h per day and 7 days every week.

The lack of significant difference in functional outcome at 3 months raises the issue of the timing of treatment decisions in poor grade aSAH patients. There was a greater proportion of patients at the weekend that were not offered treatment, and all of these were patients in poor grade. The timing of treatment in poor grade patients remains a point of debate and is being studied as part of a randomised control trial (TOPSAT II study -White et al., personal communication).

Our study was a longitudinal cohort study, adjusted for all the relevant baseline clinical characteristics and hence less prone to bias compared with a cross-sectional study. However, we did not have information about the comorbidities in patients admitted with SAH, and this is an important limitation of this study. However, the baseline demographic characteristics of those admitted on weekends and those admitted on weekdays are similar, suggesting that the distribution of comorbidities in both groups could be similar. The results from the study are derived from an old cohort. However, the NCEPOD study [1] published in September 2014 suggested that only 50 % of neurosurgical units in the United Kingdom which participated in the national survey of current practice offered weekend interventional neuro-radiological treatment. Given this report, the findings of our study are still very relevant. Furthermore, these are the only data available looking at the weekend outcomes in the United Kingdom’s NHS for patients with aSAH. We have recently instituted a United Kingdom and Ireland SAH database to record care of SAH patients and, in future, this can be used to obtain up-to-date data more easily. However, these data are not available at present.

## Conclusions

This study provides important data showing excess in-hospital mortality on weekend admissions of patients with SAH served by the United Kingdom’s NHS. This excess mortality can be partially explained by delays in access to healthcare. The lack of reproducibility of a “weekend effect” on an effect on long-term outcome needs to be studied further, ideally with a concurrent health economic analysis. The introduction of a NCEPOD recommendation for the securing of aneurysms in patients with SAH should be wary of the potential drain of medical, surgical and radiological resources without a necessary improvement in longer-term outcome.

## Electronic supplementary material

Below is the link to the electronic supplementary material.

Supplementary Figure 1 Effect of WFNS grade at admission on survival at discharge of patients with SAH (Plogrank <0.0001). Cox propotional hazard models showing effect of WFNS grade on admission and mortality in patients with acute SAH. (PNG 41 kb)
