# Supplementary figures and images for: Effect of weekend admission on in-hospital mortality and functional outcomes for patients with acute subarachnoid haemorrhage (SAH)
Source: Acta Neurochir (Wien). 2016 Mar 1;158:829–35. doi: 10.1007/s00701-016-2746-z (PMC4826657; doi:10.1007/s00701-016-2746-z)

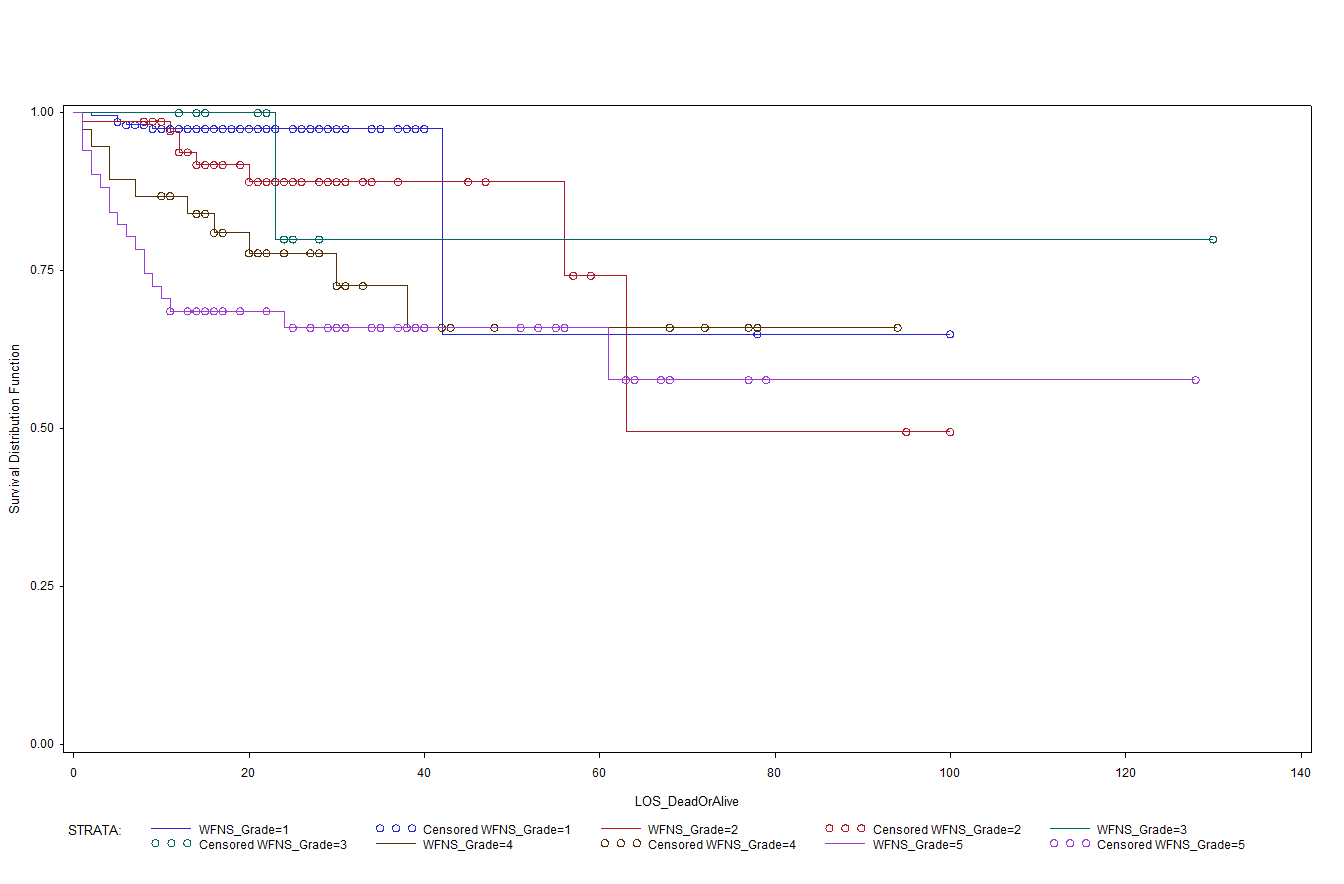

Supplement: Supplementary file 1 — Effect of WFNS grade at admission on survival at discharge of patients with SAH (Plogrank <0.0001). Cox propotional hazard models showing effect of WFNS grade on admission and mortality in patients with acute SAH. (PNG 41 kb) [file 701_2016_2746_MOESM1_ESM.png]
